# Association of sex hormones with physical, laboratory, and imaging markers of anthropometry in men and women from the general population

**DOI:** 10.1371/journal.pone.0189042

**Published:** 2018-01-11

**Authors:** Tom Seyfart, Nele Friedrich, Hanna Kische, Robin Bülow, Henri Wallaschofski, Henry Völzke, Matthias Nauck, Brian G. Keevil, Robin Haring

**Affiliations:** 1 Institute of Clinical Chemistry and Laboratory Medicine, University Medicine Greifswald, Greifswald, Germany; 2 Institute of Diagnostic Radiology and Neuroradiology, University Medicine Greifswald, Greifswald, Germany; 3 Institute for Community Medicine, University Medicine Greifswald, Greifswald, Germany; 4 German Centre for Cardiovascular Research (DZHK), University Medicine Greifswald, Greifswald, Germany; 5 Department of Clinical Chemistry, University Hospital South Manchester, Manchester, United Kingdom; 6 European University of Applied Sciences, Faculty of Applied Public Health, Rostock, Germany; University of Colorado Denver School of Medicine, UNITED STATES

## Abstract

**Objectives:**

The aim of this study was to evaluate the association of sex hormones with anthropometry in a large population-based cohort, with liquid chromatography-mass spectrometry (LCMS)-based sex hormone measurements and imaging markers.

**Study design/Main outcome measures:**

Cross-sectional data from 957 men and women from the population-based Study of Health in Pomerania (SHIP) were used. Associations of a comprehensive panel of LCMS-measured sex hormones with anthropometric parameters, laboratory, and imaging markers were analyzed in multivariable regression models for the full sample and stratified by sex. Sex hormone measures included total testosterone (TT), free testosterone (fT), estrone and estradiol, androstenedione (ASD), dehydroepiandrosterone sulfate (DHEAS), and sex hormone-binding globulin (SHBG). Domains of anthropometry included physical measures (body-mass-index (BMI), waist circumference, waist-to-height-ratio, waist-to-hip-ratio, and hip circumference), laboratory measures of adipokines (leptin and vaspin), and magnet resonance imaging-based measures (visceral and subcutaneous adipose tissue).

**Results:**

In men, inverse associations between all considered anthropometric parameters with TT were found: BMI (β-coefficient, standard error (SE): -0.159, 0.037), waist-circumference (β-coefficient, SE: -0.892, 0.292), subcutaneous adipose tissue (β-coefficient, SE: -0.156, 0.023), and leptin (β-coefficient, SE: -0.046, 0.009). In women TT (β-coefficient, SE: 1.356, 0.615) and estrone (β-coefficient, SE: 0.014, 0.005) were positively associated with BMI. In analyses of variance, BMI and leptin were inversely associated with TT, ASD, and DHEAS in men, but positively associated with estrone. In women, BMI and leptin were positively associated with all sex hormones.

**Conclusion:**

The present population-based study confirmed and extended previously reported sex-specific associations between sex hormones and various anthropometric markers of overweight and obesity.

## Introduction

The ongoing obesity epidemic is considered as one of the most serious public health problems worldwide. WHO data reports more than 1.4 billion adults to be overweight and more than half a billion adults to be obese worldwide. Thus, obesity causes 2.8 million deaths per year [1]. Furthermore, the global prevalence of obesity has nearly doubled between 1980 and 2008 [1]. In Germany, approximately 67% of men and 53% of women are overweight at present [2]. Between 1998 and 2011, the prevalence of overweight in Germany has not changed, whereas the prevalence of obesity has substantially risen, especially among men [2]. Previous investigations on health status of the German general population revealed a higher prevalence of overweight and obesity in north-east Germany, the study region of the present investigation [3].

It is well known, that overweight and obesity are strongly associated with major risk factors for increased morbidity and mortality [4]. Also, previous cross-sectional observational studies reported significant associations of sex hormones with anthropometric measures including body-mass-index (BMI) and waist-to-hip-ratio [5–7], as well as adipose tissue hormones such as leptin [8]. Additionally, meta-analyses of randomized controlled trials showed changes in anthropometry in both men and women under hormone replacement therapy, such as a decrease in fat mass [9, 10]. However, epidemiological studies to date were mostly sex-specific, or conducted within small, selected study samples only. Furthermore, sex hormones were routinely quantified by immunoassay, instead of liquid chromatography-mass spectrometry, which offer only limited precision and specificity in the low concentration range in women, and thus further limiting the reliability of previous findings.

Therefore, we addressed these limitations by investigating a population-based cohort of men and women with a comprehensive panel of liquid chromatography-mass spectrometry-measured sex hormones, including total testosterone (TT), free testosterone (fT), estrone, estradiol, androstenedione (ASD), dehydroepiandrosterone sulfate (DHEAS), and sex hormone-binding globulin (SHBG) in relation to different domains of anthropometry including physical measures such as BMI, waist circumference, waist-to-height-ratio, waist-to-hip-ratio, and hip circumference, laboratory measures such as adipose tissues cytokines leptin and vaspin, and magnet resonance imaging (MRI)-based measures such as visceral adipose tissue and subcutaneous adipose tissue.

## Materials and methods

### Study population

**Fig 1:**
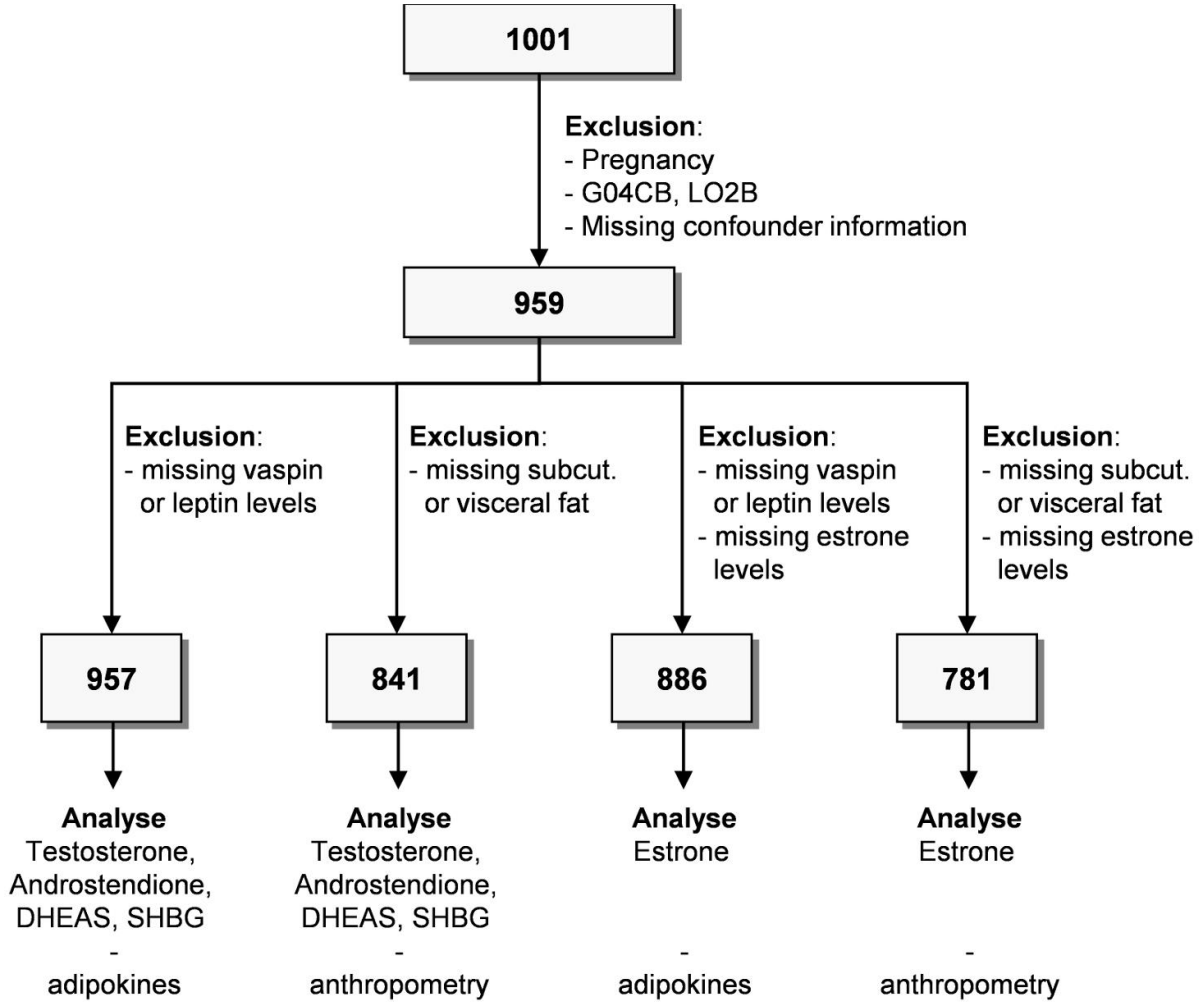
Flow chart of the study sample. DHEAS, dehydroepiandrosterone-sulfate; SHBG, sex hormone-binding globulin; Anatomical Therapeutic Chemical Classification System Code for Urologicals: LO2B and for Testosterone-5-alpha-reductase-inhibitors: G04CB.

Data from the Study of Health in Pomerania (SHIP) were used. SHIP is a population-based cohort study focusing on an area in northeastern Germany. Study design and sampling methods were described previously [11]. 8,826 individuals with an age of 20–79 years with German citizenship and main residency in the study area were invited. 4,420 individuals (2,145 men) participated (response 50.1%) in baseline examinations of SHIP-TREND. Examinations were conducted between September 2008 and September 2012. A subsample of 1001 subjects was used for this study. Of these subjects we excluded men or women with the following conditions (overlap exists): self-reported bilateral oophorectomy (n = 29), intake of testosterone 5a-reductase inhibitors (Anatomical Therapeutic Chemical (ATC) classification: G04CB; n = 1) or sex hormone antagonists (ATC L02B; n = 4) and missing covariate or outcome data (n = 2). Furthermore, all subjects with missing sex hormone, leptin or anthropometric measurements were excluded resulting in different study populations ranging from 781 to 957 subjects (Fig 1). All participants gave informed written consent prior to examination. This study protocol complies with the Declaration of Helsinki, and was approved by the Ethics Committee of the University of Greifswald.

### Anthropometric measurements

Weight was measured (to the nearest 0.1 kg) with standard digital scales without shoes and in light clothing only. Waist circumference was measured (to the nearest 0.1 cm) midway between the lower rib margin and the iliac crest in horizontal plane with a tape measure. Height was measured (to the nearest 0.1 cm) with a digital ultrasound instrument. BMI was calculated from the body weight in kilograms and height in meters [BMI = kg / m^2^].

### Sex hormone measurements

Fasting blood samples were taken from the cubital vein before noon, and prepared for either instant analysis or for storage at -80°C for further analysis. As previously described, TT, ASD, and estrogens were measured from frozen aliquots with liquid chromatography-mass spectrometry [12]. For TT and ASD, the standard curve was linear up to 50.0 nmol/L. 0.25 nmol/L was the lower limit of quantitation. Intra- and inter-assay coefficients of variation were <10% for the range between 0.3–35 nmol/L. For estrone and estradiol the measurement range was 25–2000 pmol/L. The lower limit of detection was 3.9 and 8.0 pmol/L. Inter-assay imprecisions were 5.3, 3.8 and 5.1% for estrone and 5.4, 3.7 and 4.9% at concentrations of 125, 400 and 1500 pmol/L for estradiol. Intra-assay imprecisions were 4.0, 3.4 and 5.0% for estrone and 3.1, 3.5 and 4.0% for estradiol for these concentrations, respectively. SHBG and DHEAS were measured with chemiluminescent immunoassay Immulite 2000 XPi (Siemens Healthcare Diagnostics, Eschborn, Germany) with an inter-assay coefficient of variation of 3.5% and 8.3% at low level, and 4.8% and 5.4% at high level, respectively. FT was calculated from measured TT and SHBG: [$\text{fT}\left(\text{nmol}/\text{L}\right)=\left(\left(-\text{a}+\sqrt{b}\right)/\text{c}\right)/10^{-9}$ with a = SHBG (nmol/L) -TT (nmol/L) +23.43, b = a^2^ + (4*23.43*TT (nmol/L))] and c = 2*23.43*10^9^ for a standard average albumin concentration of 4.3 g/dL.

### Abdominal adipose tissue MRI measurements

Standardized whole body MRI was performed on a 1.5-T MR system (Magnetom Avanto; Siemens Medical Systems, Erlangen, Germany including a T1-weighted, two-point Dixon volume interpolated breath-hold exam (VIBE) sequence at three stations covering the full abdomen [13]. The present study analyzed unenhanced multi-echo VIBE sequences acquired with a repetition time (TR) of 7.5 (ms), echo times (TE) of 2.4/4.8 (ms), 10° flip angle with a voxel size of 1.64 x 1.64 x 4.0 mm and a slice gap of 0.8 mm. The MRI sequences were acquired using a field of view, 420 × 289 mm; matrix, 256 × 120; bandwidth of 290 and 300 Hz per pixel and parallel imaging (generalized auto-calibrating partially parallel acquisition) with an effective acceleration factor of 2.0. Each station data set was acquired in the axial plane during a single 19-second breath hold.

**Fig 2:**
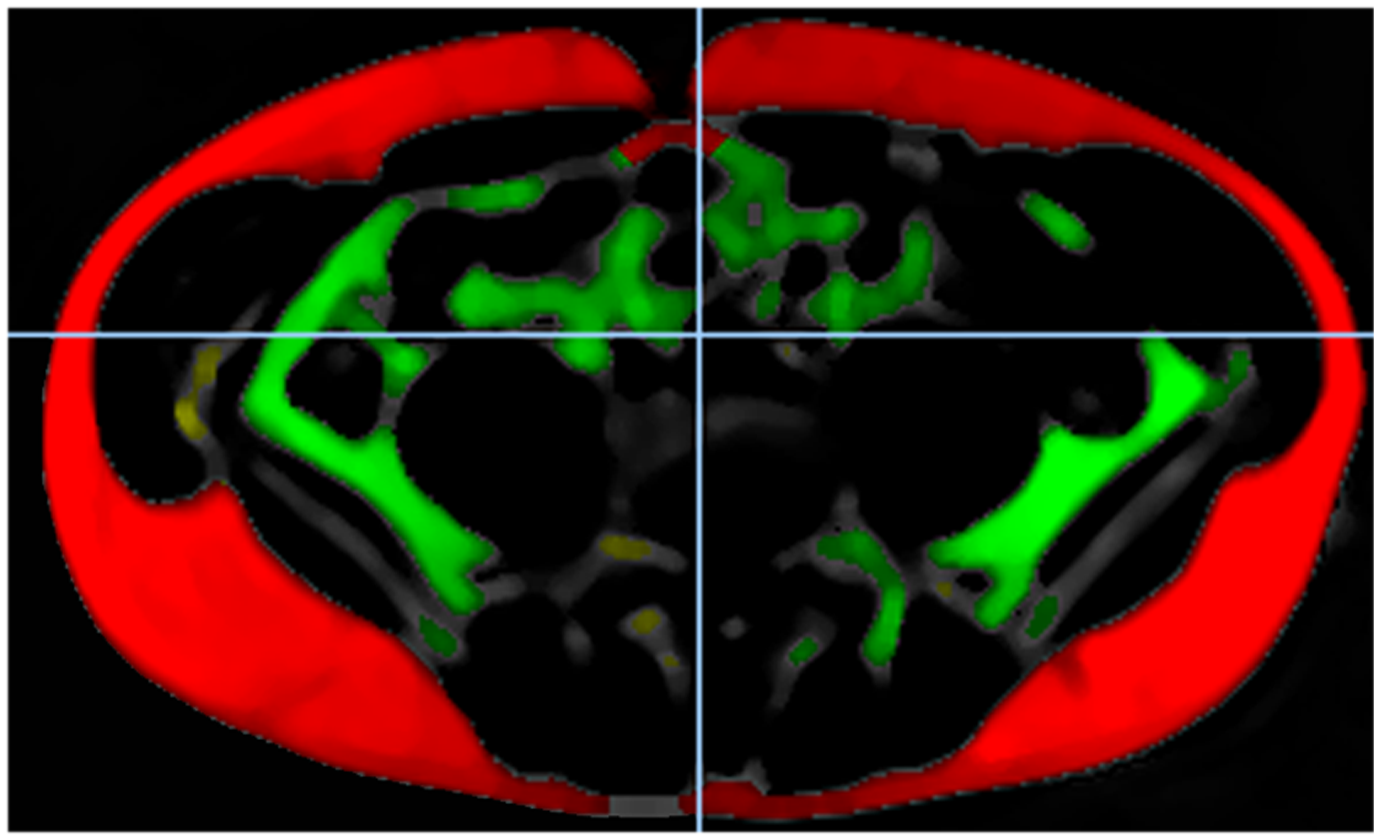
MRI post-processing figure depicting the fat quantification pipeline. Subcutaneous adipose tissue is highlighted red, visceral adipose tissue is highlighted green, other fat tissue is highlighted yellow.

The quantification of subcutaneous and visceral adipose tissue was performed with the automatic tissue and labeling analysis software ATLAS and an in-house developed software from the University of Ulm [14]. Thus, three stations were composed according to the magnetic resonance table position. The semiautomated image analysis comprised a reading consisting of ATLAS quantification and a subsequent manual correction applied from a medical doctor and a medical student after certification. Adipose tissue of both arms and breast tissue were excluded from quantitative image analysis. Intra- and inter-observer were calculated in a random subsample of 5%. The intra-observer variability was given as mean and 1.96 standard deviation and ranges from -0.24 ± 3.37% to 1.75 ± 4.98% for the subcutaneous adipose tissue and from -0.59 ± 2.08% to 1.47 ± 3.19% for the visceral adipose tissue. The inter-observer variability was 1.59 ± 4.54% for subcutaneous adipose tissue and 0.56 ± 3.15% for the visceral adipose tissue. Intra-class-correlation ranges was 0.998 for subcutaneous and 0.999 for visceral adipose tissue. Fig 2 presents the MRI post-processing, depicting the fat quantification pipeline.

### Covariables

To assess socio-demographic and behavioral characteristics, a computer-assisted personal interview was conducted. Data on sex, age, alcohol consumption, and physical training were acquired, as well as data on medical history including pregnancy, gynecological surgery, bilateral oophorectomy, and medication use. Beverage-specific pure ethanol volume proportions were used to evaluate mean daily alcohol consumption. Physical inactivity was defined as less than one hour of physical training per week during winter or summer. Regarding smoking habits, every participant was placed in one of three categories: current, former, and never-smoker.

Systolic and diastolic blood pressure were measured after a resting period of at least five minutes on the right arm of seated subjects using an oscillometric digital blood pressure monitor (HEM-705CP, Omron Corporation, Tokyo, Japan). Three measurements were performed with an interval between the readings of three minutes. The mean of the second and third measurement was used. Hypertension was defined as blood pressure ≥140/90 mmHg or use of antihypertensive medication (ATC codes C02, C03, C04, C07, C08, C09) [15].

A summative score consisting of diagnosis of angina pectoris, peripheral artery disease, heart failure, stroke, and/or myocardial infarction was used to define cardiovascular disease. Self-reported physician’s diagnosis, use of antidiabetic medication (ATC code A10) and/or HbA1c ≥6.5% and <20% were used to define type 2 diabetes mellitus. Waist circumference ≥120 cm, blood pressure ≥130/85 mmHg or self-reported antihypertensive drug treatment, non-fasting glucose ≥6.1 mmol/l or antidiabetic treatment (ATC codes A10A, A10B), non-fasting triglycerides ≥1.7 mmol/ l or lipid-lowering treatment (ATC codes C10AB, A10AD), and/or high-density lipoprotein cholesterol ≤1.3 mmol/l were used for the assessment of metabolic syndrome and are premised on the Joint Scientific Statement to harmonize metabolic syndrome [16]. Self-reported physician’s diagnoses were used to define dyslipidemia and/or cancer. Dimension Vista 500 analytical system (Siemens Healthcare Diagnostics GmbH, Eschborn, Germany) was used to measure total cholesterol and high-density lipoprotein cholesterol. Skilled technical personnel performed all assays according to the manufacturers’ recommendations.

Women were stratified into pre- and post-menopausal applying a previously published categorization: Women aged between 40 and 60 years or <40 years of age who reported menstrual cycle were classified as pre-menopausal. Women aged between 40 and 60 years and women ≥ 60 years of age who reported no menstrual cycle were classified as post-menopausal [17]. Use of oral contraceptive (G03A) and/or hormone therapy (G03C, G03D, or G03F) was assessed based on ATC codes [12].

### Statistical analysis

Categorical data are given as percentage; continuous data are given as mean (standard deviation) or median (p25^th^, p75^th^). For bivariate comparisons the Kruskal-Wallis test (continuous data) or χ2-test (nominal data) were used to compare men and women. All models were stratified by sex. Analyses of variance (ANOVA) and multivariable quantile regression models were performed to estimate the independent associations of sex hormones (exposure variables) as either categorical or continuous variables with anthropometric markers as well as leptin levels (outcome variables). In ANOVA, exposure variables were categorized into three groups according to their tertiles. To detect possible nonlinear associations, quantile regression models with restricted cubic splines with three knots pre-specified located at the 5th, 50th and 95th percentile were compared by likelihood ratio test to the fit of a linear model. ANOVA models were adjusted for age, sex, smoking and physical inactivity. Quantile regression models were additionally adjusted for diabetes, hypertension, and cholesterol. A p-value of < 0.05 was considered statistically significant. Statistical analyses were performed with SAS 9.4 (SAS Institute Inc., Cary, NC, USA).

## Results

Table 1 presents the baseline characteristics of the full study sample, stratified by sex.

**Table 1:** Baseline characteristics of the study population, stratified by sex.

Variable	Women (N = 520)	Men (N = 437)	P
Age, years	50.0 (41.0; 59.0)	50.0 (40.0; 60.0)	0.90
Total testosterone, nmol/L	0.8 (0.6; 1.0)	17.4 (14.5; 20.7)	< 0.01
Androstenedione, nmol/L	2.3 (1.7; 3.4)	2.8 (2.1; 3.7)	< 0.01
DHEAS, mg/L	1.0 (0.7; 1.5)	1.7 (1.0; 2.5)	< 0.01
Estrone, nmol/L	119.5 (72.8; 228.0)	114.5 (94.3; 145.8)	0.34
Estradiol, nmol/L	216.8 (70.9; 429.5)	76.8 (60.6; 91.2)	< 0.01
SHBG, nmol/L	55.7 (42.1; 79.5)	36.1 (29.3; 45.7)	< 0.01
Leptin, ng/ml	17.8 (10.9; 28.1)	6.2 (3.5; 9.2)	< 0.01
Body mass index, kg/m^2^	25.9 (23.1; 29.3)	27.6 (25.0; 29.9)	< 0.01
Waist circumference, cm	80.5 (73.8; 89.0)	94 (86.2; 101.5)	< 0.01
Waist-to-hip-ratio	0.82 (0.78; 0.86)	0.93 (0.88; 0.97)	< 0.01
Subcutaneous fat, L	8.0 (6.1; 10.8)	6.1 (4.7; 8.1)	< 0.01
Visceral fat, L	2.4 (1.7; 3.6)	4.9 (2.9; 6.9)	< 0.01
Current smoking, %	22.1	22.9	< 0.01
Physical inactive, %	26.4	27.0	0.82
Oral contraceptive use, %	15.4	-	-
Hormone replacement therapy, %	5.0	-	-
Hypertension, %	66.7	56.1	< 0.01
T2DM, %	0.8	2.1	0.09
Metabolic syndrome, %	16.4	26.8	< 0.01
Systolic blood pressure, mmHg	116.5 (107.5; 127.5)	129.5 (121.0; 140.0)	< 0.01
Diastolic blood pressure, mmHg	74.0 (68.5; 79.5)	78.5 (72.5; 85.0)	< 0.01
Total cholesterol, mmol/L	5.5 (4.9; 6.2)	5.4 (4.6; 6.1)	0.02
Total triglycerides, mmol/L	1.4 (0.8; 1.6)	1.3 (0.9; 1.9)	< 0.01
Serum glucose, mmol/L	5.1 (4.9; 5.5)	5.4 (5.1; 5.8)	< 0.01
HbA1c, %	5.1 (4.8; 5.5)	5.2 (4.9; 5.6)	< 0.01

Continuous data are expressed as median (25th and 75th percentiles; nominal data are given as percentages. χ2-test (nominal data) or Kruskal-Wallis test (interval data) were performed. DHEAS, dehydroepiandrosterone-sulfate; HbA1c, glycated hemoglobin A1c; SHBG, sex hormone-binding globulin; T2DM, type 2 diabetes mellitus.

With respect to anthropometric parameters women had lower BMI, waist-circumference, waist-to-hip-ratio, and visceral adipose tissue, but higher subcutaneous adipose tissue compared to men.

**Fig 3:**
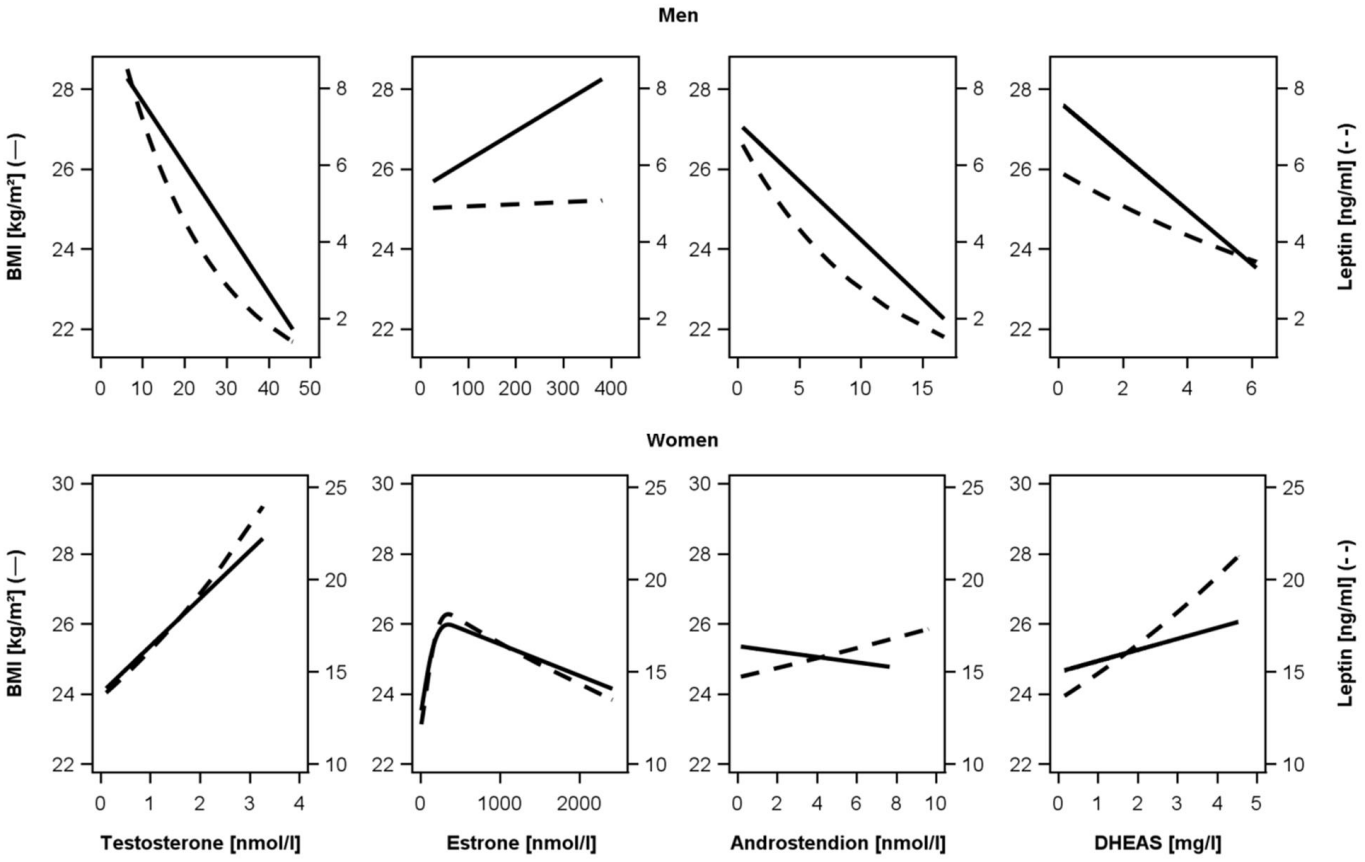
Associations of sex hormones with leptin. Analysis of testosterone, estrone, androstendione, and dehydroepiandrosterone-sulfate (DHEAS) with body-mass-index (BMI) and leptin among men (upper part) and women (lower part). Linear regression adjusted for age, sex, smoking, physical activity, type 2 diabetes mellitus, hypertension, and cholesterol.

Regarding laboratory measures women had lower TT, ASD, and DHEAS, but higher estrone, estradiol, SHBG, and leptin. Results of the multivariable linear regression analyses are presented in Table 2 and Fig 3.

**Table 2:** Association between sex hormones and anthropometry in men and women.

	MEN					
	Body-mass-index		Waist circumference		WHR	
	Beta (SE)	p	Beta (SE)	p	Beta (SE)	p
Androstendione	-0.294 (0.112)	0.01	-0.892 (0.292)	< .01	-5.37E^-03^ (3.22E^-03^)	0.10
DHEAS	-0.680 (0.197)	0.00	-1.460 (0.696)	0.04	0.022 (0.014)	0.11
Estrone	7.22E^-03^ (4.61E^-03^)	0.12	0.016 (0.011)	0.15	-4.98E^-03^ (2.19E^-03^)	0.02
SHBG	-0.075 (0.013)	< .01	-0.184 (0.035)	< .01	-1.01E^-05^ (4.34E^-05^)	0.82
Testosterone	-0.159 (0.037)	< .01	-0.448 (0.077)	< .01	-9.11E^-04^ (2.59E^-04^)	< .01
	Subcutaneous fat		Visceral fat		Log(Leptin)	
	Beta (SE)	p	Beta (SE)	p	Beta (SE)	p
Androstendione	-0.260 (0.065)	< .01	-0.125 (0.093)	0.18	-0.090 (0.018)	< .01
DHEAS	-0.266 (0.163)	0.10	-0.150 (0.122)	0.22	-0.085 (0.027)	0.00
Estrone	-2.98E^-03^ (3.62E^-03^)	0.41	5.83E^-03^ (3.60E^-03^)	0.11	1.11E^-04^ (9.94E^-04^)	0.91
SHBG	-0.054 (0.010)	< .01	-0.060 (0.007)	< .01	-0.016 (0.003)	< .01
Testosterone	-0.156 (0.023)	< .01	-0.145 (0.022)	< .01	-0.046 (0.009)	< .01
	WOMEN					
	Body-mass-index		Waist circumference		WHR	
	Beta (SE)	p	Beta (SE)	p	Beta (SE)	p
Androstendione	-0.077 (0.206)	0.71	0.176 (0.496)	0.72	1.82E^-03^ (2.90E^-03^)	0.53
DHEAS	0.316 (0.404)	0.43	0.706 (0.901)	0.43	0.014 (0.005)	0.00
Estrone	0.014 (0.005)	< .01	6.47E^-03^ (4.29E^-03^)	0.13	1.36E^-05^ (1.19E^-05^)	0.26
Estrone'	-1.98E^-07^ (7.27E^-08^)	0.01	-	-	-	-
SHBG	-0.132 (0.031)	< .01	-0.333 (0.074)	< .01	-1.30E^-03^ (3.64E^-04^)	< .01
SHBG'	2.28E^-05^ (7.18E^-06^)	< .01	5.63E^-05^ (1.69E^-05^)	< .01	1.87E^-07^ (8.50E^-08^)	0.03
Testosterone	1.356 (0.615)	0.03	1.639 (1.296)	0.21	-2.05E^-03^ (8.43E^-03^)	0.81
	Subcutaneous fat		Visceral fat		Log(Leptin)	
	Beta (SE)	p	Beta (SE)	p	Beta (SE)	p
Androstendione	-0.185 (0.194)	0.34	0.056 (0.052)	0.28	0.017 (0.031)	0.58
DHEAS	0.419 (0.309)	0.18	0.142 (0.104)	0.17	0.101 (0.054)	0.06
Estrone	9.80E^-03^ (4.36E^-03^)	0.03	6.45E^-05^ (5.07E^-04^)	0.90	2.25E^-03^ (8.45E^-04^)	0.01
Estrone'	-1.41E^-07^ (7.26E^-08^)	0.05			-3.17E^-08^ (1.30E^-08^)	0.02
SHBG	-0.121 (0.023)	< .01	-0.064 (0.010)	< .01	-0.016 (0.004)	< .01
SHBG'	1.97E^-05^ (5.34E^-06^)	< .01	1.12E^-05^ (2.13E^-06^)	< .01	2.23E^-06^ (7.85E^-07^)	< .01
Testosterone	0.549 (0.527)	0.30	0.247 (0.164)	0.13	0.174 (0.091)	0.06

Data are beta-coefficients (Beta) and standard error (SE) with p-values. Linear regression model were adjusted for age, sex, smoking, physical activity, diabetes, hypertension, and cholesterol. DHEAS, dehydroepiandrosterone-sulfate; SHBG, sex hormone-binding globulin; WHR, waist-to-hip-ratio.

**Fig 4:**
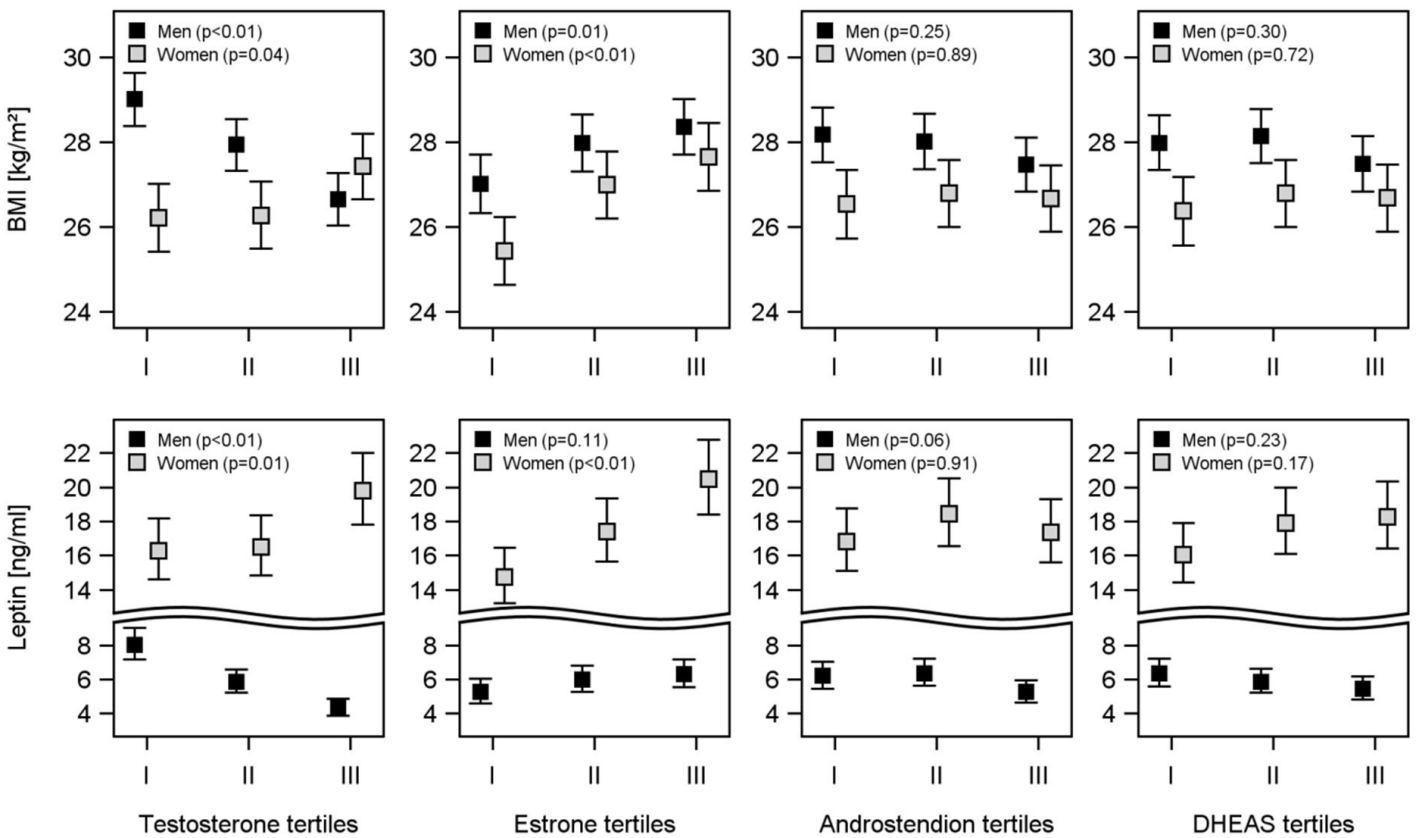
Associations of sex hormones with BMI and leptin levels. Estimated means of the body-mass-index (BMI) and leptin by tertiles of testosterone, estrone, androstenedione, and dehydroepiandrosterone-sulfate (DHEAS). Analysis of variance was adjusted for adjusted for age, sex, smoking, and physical activity.

In men inverse associations between all considered anthropometric parameters with TT were found: BMI (β-coefficient, standard error (SE): -0.159, 0.037), waist-circumference (β-coefficient, SE: -0.892, 0.292), waist-to-hip-ratio (β-coefficient, SE: -9.11E^-04^, 2.59E^-04^), subcutaneous adipose tissue (β-coefficient, SE: -0.156, 0.023), visceral adipose tissue (β-coefficient, SE: -0.145, 0.022), and leptin (β-coefficient, SE: -0.046, 0.009). Similarly, ASD was inversely associated with BMI (β-coefficient, SE: -0.294, 0.112), waist-circumference (β-coefficient, SE: -0.892, 0.292), subcutaneous adipose tissue (β-coefficient, SE: -0.260, 0.065), and leptin (β-coefficient, SE: -0.090, 0.018) in men. Furthermore, DHEAS was inversely associated with BMI (β-coefficient, SE: -0.680, 0.197), waist-circumference (β-coefficient, SE: -1.460, 0.696), and leptin (β-coefficient, SE: -0.085, 0.027) in men. Additionally, SHBG was inversely associated with BMI (β-coefficient, SE: -0.075, 0.013), waist-circumference (β-coefficient, SE: -0.184, 0.035), subcutaneous adipose tissue (β-coefficient, SE: -0.054, 0.010), visceral adipose tissue (β-coefficient, SE: -0.145, 0.022), and leptin (β-coefficient, SE: -0.046, 0.009) in men. No significant association between estrone and anthropometric parameters were found in men. In women, TT was positively associated with BMI (β-coefficient, SE: 1.356, 0.615). Estrone was positively associated with BMI (β-coefficient, SE: 0.014, 0.005), subcutaneous adipose tissue (β-coefficient, SE: 9.80E^-03^, 4.36E^-03^), and leptin (β-coefficient, SE: 2.25E^-03^, 8.45E^-04^) in women. SHBG was inversely associated with BMI (β-coefficient, SE: -0.132, 0.031), waist-circumference (β-coefficient, SE: -0.333, 0.074), waist-to-hip-ratio (β-coefficient, SE: 1.30E^-03^, 3.64E^-04^), subcutaneous adipose tissue (β-coefficient, SE: -0.121, 0.023), visceral adipose tissue (β-coefficient, SE: -0.064, 0.010), and leptin (β-coefficient, SE: -0.016, 0.004) in women. Similarly, we observed these results in ANOVA (Fig 4): In men, TT, ASD, and DHEAS were inversely associated with BMI and leptin, whereas estrone was positively associated with BMI and leptin. In women, all sex hormones were positively associated with BMI and leptin in ANOVA (Fig 4).

## Discussion

The present cross-sectional study investigated associations of sex hormones and anthropometric markers in men and women from the general population.

With regard to androgens, we observed, that TT was inversely associated with all anthropometric parameters and leptin in men, whereas TT was positively associated with BMI in women. Our results are in line with previous cross-sectional observational studies, which have revealed inverse associations of TT with multiple anthropometric markers in men, in particular with BMI and waist-to-hip-ratio [5–7]. This effect has been linked to high serum estrogens, generated by aromatization of testosterone in adipose tissue [18], which in turn suppress gonadotropin-releasing hormone and luteinizing hormone, thereby suppressing testicular testosterone production [19]. A recently published study revealed a causal effect of BMI on serum testosterone in men [20]. According to these authors, population level interventions to reduce BMI are expected to increase serum testosterone in men.

With regard to estrogens, the presently observed positive associations with BMI, subcutaneous adipose tissue and leptin in women are in line with previous studies [7, 21]. As previously reported obese men not only showed increased serum estrogens [18], but also decreased concentrations of SHBG, and therefore leading to a further increase in free estrogens not bound to SHBG [21, 22].

Likewise, considering SHBG, our results showed that SHBG was inversely associated with all anthropometric parameters and leptin, in men and women, and also with vaspin in women. Previous studies revealed inverse associations of serum SHBG with multiple anthropometric parameters, such as BMI (5), subcutaneous adipose tissue, and waist-circumference [7]. The effect of low SHBG in increased obesity risk has been previously described [21, 22].

Regarding adipose tissue, previous studies showed, that this tissue produces adipokines and plays a key role in regulation of energy metabolism and influences sex hormone conversion [23]. Our results showed that testosterone was inversely associated with the adipokine leptin in men and leptin also being generally higher in women, which is in line with previous studies [8].

The effects of sex hormones on anthropometric parameters have previously been investigated in studies on hormone replacement therapy. Meta-analyses of randomized controlled trials showed beneficial effects for hormone replacement therapy with estrogens in women [9] and with testosterone in men [10]. For testosterone therapy in men, these effects include a decrease in body fatness measures such as total body fat, and subcutaneous adipose tissue, and an increase in fat free mass, muscle size and strength in healthy men as well as in hypogonadal men [10]. In the same way, a meta-analysis showed that estrogen therapy increased lean body mass, reduced waist circumference and abdominal fat in post-menopausal women [9]. However, a systematic review and meta-analysis showed adverse effects for testosterone therapy in men, such as an increase in hemoglobin, hematocrit, and a decrease in high-density lipoprotein cholesterol [24].

An overweight and obesity-related risk factor burden and morbidity has been observed for a wide range of non-communicable diseases. Previous studies showed that overweight and obesity are significantly associated with hypertension, cardiovascular disease, type 2 diabetes mellitus, stroke, high cholesterol, asthma, arthritis, specific cancers, and poor health status in general [4]. In the same manner as overweight and obesity-related anthropometric parameters, sex hormones have previously been shown to serve as biomarkers and thus predict disease- and mortality-risk: Studies conducted on this subject suggested low serum testosterone in men as a potential biomarker for an increased risk of all-cause mortality [25], increased risk of prevalent and incident metabolic syndrome [26], increased risk of type 2 diabetes mellitus [27], and increased cardiovascular risk [28]. However, a population-based Mendelian randomization analysis revealed no evidence for causal associations of testosterone with cardio-metabolic risk factors or mortality: This study suggested that previously reported observational associations might have resulted from residual confounding or reverse causation [29]. Furthermore, previous studies also suggested low SHBG as a predictor for cardio-metabolic morbidity, metabolic syndrome and type 2 diabetes mellitus [26, 30]. In this respect, the specific mechanisms linking metabolic and male hypogonadism have not been completely clarified to date. Low testosterone could be considered as one of many adverse consequences of overweight and obesity. On the other hand, according to a meta-analysis, hypogonadism could contribute to the accumulation of excess fat and to the reduction of insulin-sensitive muscular mass [31]. Thus, for the prevention of diseases, assessment of sex hormones, especially testosterone in young and middle-age men, may allow early interventions in the general population [32].

Strengths of the present study include the large population-based sample, quantification of sex hormones by liquid chromatography-mass spectrometry, a broad assessment of anthropometric markers including those measured from MRI, and a high level of quality assurance, particularly in standardization of non-invasive examination methods and data management. Potential limitations may arise from the cross-sectional design, allowing no conclusions regarding causality. The generalizability of our findings is limited due to the exclusively Caucasian study sample. Furthermore, potential misclassification bias from self-reported health data might have reduced the external validity of our findings. Blood samples were taken throughout the day. However, a previous investigation in SHIP showed only minor differences in total testosterone levels between serum samples drawn before midday and afternoon, therefore, this variation is expected to be minimal [33]. Finally, the present findings may be limited by androgen measurements based on blood samples taken during any phase of the menstrual cycle. Since we did not assess the phase of the menstrual cycle, we were not able to adjust for this potential source of bias.

## Conclusion

In conclusion, the present population-based study extended previously reported sex-specific associations between sex hormones and various anthropometric markers of overweight and obesity. TT was inversely associated with all considered anthropometric parameters in men, whereas all sex hormones were positively associated with BMI and leptin in women. These sex-specific differences should be noted in establishing reference ranges and clinical cut-off points, further studies regarding sex hormones as biomarkers for overweight and obesity, and to evaluate individual overweight and obesity-related risk factor burden. Future research from prospective cohort studies, as well as interventional trials, is needed to investigate the molecular mechanisms of these associations and to assess their causal direction.
